# *Streptococcus oralis* induces platelet aggregation through coordinated GPIbα and FcγRIIA signalling

**DOI:** 10.1080/09537104.2026.2688088

**Published:** 2026-10-29

**Authors:** Rachel Lamerton, Amy McMahon, Adam O’Neill, Samantha King, Steven W. Kerrigan

**Affiliations:** 1 Cardiovascular Infection Research Group, School of Pharmacy and Biomolecular Sciences, RCSI University of Medicine and Health Sciences, Dublin 2, Ireland;; 2 Center for Microbe and Immunity Research, The Abigail Wexner Research Institute at Nationwide Children’s Hospital, Columbus, OH, USA;; 3 Department of Pediatrics, The Ohio State University, Columbus, OH, USA

**Keywords:** Antibody, FcγRIIa, GPIbα, infective endocarditis, platelets, sialic acid, *Streptococcus oralis*

## Abstract

**Background:**

*Streptococcus oralis* is the most prevalent viridans group streptococcus associated with infective endocarditis (IE), yet the platelet activation mechanisms that underpin its pathogenicity remain poorly defined. Platelet–bacterium interactions are central to vegetation formation, but the relative contributions of direct adhesion and antibody-dependent signaling in *S. oralis*-induced platelet aggregation are unclear.

**Objectives:**

To define the molecular mechanisms by which *S. oralis* induces platelet activation and aggregation, and to determine how bacterial adhesins and host factors cooperate to drive this response.

**Methods:**

Platelet aggregation and dense granule secretion were assessed using light transmission aggregometry and ATP release assays in platelet-rich plasma and washed platelets. An IE isolate of *S. oralis* and isogenic mutants lacking functional Fap1 were used alongside pharmacological and antibody-based inhibition of platelet receptors and signaling pathways.

**Results:**

*S. oralis* ATCC 10557 induced robust platelet aggregation with a characteristic lag phase and strong dense granule release. Aggregation required integrin αIIbβ3 signaling and was abolished by inhibition of glycoprotein Ibα (GPIbα) or FcγRIIA. Loss of the serine-rich repeat protein Fap1, or mutation of its conserved sialic acid-binding motif, abrogated platelet aggregation. While most donors required a plasma component for aggregation, supplementation of washed platelets with human IgG fully restored aggregation in a dose-dependent manner. Importantly, IgG was unable to bypass the requirement for Fap1–GPIbα interaction, indicating that direct bacterial adhesion is an essential initiating step that enables antibody-dependent FcγRIIA signaling. Substantial inter-individual variability was observed in aggregation kinetics and IgG dependence.

**Conclusions:**

*S. oralis* induces platelet aggregation through a dual, non-redundant mechanism requiring Fap1-mediated engagement of GPIbα and IgG-dependent FcγRIIA signaling. This cooperative pathway drives potent platelet activation and exhibits marked host-dependent variability. These findings provide new mechanistic insight into early events in IE pathogenesis and highlight platelet–bacterium interactions as potential targets for risk stratification and therapeutic intervention.

## PLAIN LANGUAGE SUMMARY

**What is the context?:**

- Infective endocarditis is an infection that causes clumps of bacteria, platelets and other blood components to form on the inside of the heart. These clumps frequently form on the heart valves making it hard for them to open and close so the heart struggles to beat normally. There were over 1 million cases worldwide in 2021, and the infection is fatal in about 25% of cases. It is difficult to treat, usually requiring weeks of intravenous antibiotic treatment in hospital, and about 50% of cases need surgery in addition to the antibiotics.
- We investigated how a bacteria called *Streptococcus oralis* (*S. oralis*) that can cause infective endocarditis causes platelets to clump together.

**What is new?:**

- We show that for *S. oralis* to cause platelets to clump together and form a blood clot, two specific platelet receptors (called GPIbα and FcγRIIA), and antibodies to *S. oralis*, are needed in the majority of people.
- We show that the protein on the outside of *S. oralis* that interacts with platelets is called Fap1. If you remove Fap1, platelets no longer clump together in response to the bacteria.
- We also found that there are groups of people whose platelets react differently to most in response to *S. oralis*, either by being much slower to clump together, or by being able to clump together without the FcγRIIA receptor and antibody.

**What is the impact?:**

- Understanding how bacteria and platelets interact to form these clumps means we can direct future research into them as targets for new treatments for infective endocarditis.
- Identifying groups of people who respond differently to the bacteria means personalized medicine approaches could be taken in the future.

## Introduction

Infective endocarditis (IE) is a life-threatening condition characterized by the colonization of endocardial surfaces, prosthetic cardiac valves or implanted cardiac devices by microbial pathogens, leading to the formation of vegetative thrombi composed of fibrin, platelets, and immune cells.^1^ Despite advances in diagnostic techniques and antimicrobial therapy, IE continues to carry high morbidity and mortality rates,^2,3^ particularly in cases involving prosthetic valves, intracardiac devices, or immunocompromised patients.^4^ Early events that drive vegetation formation are central to disease pathogenesis, yet remain incompletely understood, limiting the development of targeted therapeutic or preventative strategies.

Staphylococcal and streptococcal species account for the majority of IE cases.^5^ Among these, oral viridians group streptococci (VGS) are frequently associated with subacute disease, with *Streptococcus oralis* one of the most prevalent VGS species isolated from IE, followed by *Streptococcus sanguinis* and *Streptococcus gordonii*.^6,7^ These organisms gain transient access to the bloodstream during routine activities such as dental procedures, tooth brushing, or oral mucosal injury. Once circulating, they preferentially adhere to sites of endothelial damage, initiating the cascade of events that culminates in vegetation formation.^1^

Platelets play a central role in this process. Beyond their established function in hemostasis, platelets act as innate immune sentinels, rapidly responding to endothelial injury and microbial invasion.^8^ In IE, platelet activation and aggregation at sites of bacterial adherence provide the structural scaffold for vegetations while simultaneously shielding bacteria from immune clearance and antimicrobial killing.^1^ These platelet–bacterial interactions can therefore promote microbial persistence and contribute directly to disease progression, embolic complications, and valve destruction.

Extensive work has characterized platelet interactions with *S. sanguinis* and S. *gordonii*. These studies have identified bacterial serine-rich repeat proteins (SRRPs) as key adhesins that bind sialylated glycans on GPIbα.^9–12^ Subsequent platelet aggregation requires integrin αIIbβ3 (GPIIb/IIIa) engagement and is amplified by immunoglobulin G (IgG) binding to FcγRIIA.^13–16^ Bacterial-induced platelet aggregation is typically associated with a characteristic lag phase, distinct from classical platelet agonists.^17^ Short lag times (<5 min) are generally indicative of direct platelet–bacterium interactions, whereas prolonged lag times suggest indirect mechanisms involving plasma factors such as complement.^18^ For example, *S. sanguinis* strain 133–79 induces rapid, GPIbα-dependent aggregation,^19^ whereas strain NCTC 7863 triggers delayed aggregation that requires complement activation.^20,21^

Despite its disproportionate contribution to IE burden, the mechanisms underpinning S. *oralis*-induced platelet aggregation remain comparatively poorly defined. Previous studies have demonstrated a requirement for platelet GPIbα in *S. oralis* adhesion^22^ and FcγRIIA in aggregation,^23,24^ but were unable to identify the bacterial surface protein(s) responsible. More recently, static adhesion assays have shown that the *S. oralis* SRRP Fap1 binds sialic acid residues on platelets,^24^ suggesting a potential mechanism for platelet engagement. However, whether Fap1-mediated adhesion is sufficient to drive platelet aggregation, and how this interaction integrates with FcγRIIA-dependent signaling, remains unknown.

Consequently, the relative contributions of GPIbα, GPIIb/IIIa, and FcγRIIA to *S. oralis*-induced platelet aggregation, and the extent to which these pathways operate independently or synergistically during early infection, have not been systematically explored. Addressing this gap is critical to understanding how *S. oralis* promotes vegetation formation and why platelet responses vary markedly between individuals.

In this study, we comprehensively characterize the mechanisms underlying *S. oralis*-induced platelet aggregation using an IE isolate. We demonstrate that platelet activation requires a dual mechanism involving Fap1-mediated engagement of GPIbα and antibody-dependent FcγRIIA signaling, and we define how these pathways cooperate to drive robust aggregation. Together, these findings provide new insight into the early platelet–bacterium interactions that underpin IE pathogenesis and highlight host- and pathogen-specific factors that may influence disease susceptibility.

## Methods

### Bacterial strains, culture and preparation

The strains used in this study (*S. oralis* ATCC 10557 Sm^r^, ATCC 10557 Fap1 Janus and ATCC 10557 Fap1R489E) were generated previously.^25^ ATCC 10557 Sm^r^ is the parental strain that contains a point mutation in *rpsL*(K56T) that allowed generation of the unmarked point mutant using the two-step Janus cassette system.^26^

Bacteria were routinely cultured from glycerol stocks in 10 ml Todd-Hewitt Broth supplemented with 0.2% yeast extract (THY) overnight at 37°C. As appropriate, broth was supplemented with 500 μg/ml kanamycin or 200 μg/ml streptomycin.^25^ Bacteria were harvested and washed twice by centrifuging at 3800 *g* for 5 min, the pellet was resuspended in phosphate buffered saline (PBS). At the final wash, bacteria were resuspended in PBS to an optical density of 600 nm (OD_600_) = 1.6.

### Blood collection and ethics

Ethical approval was obtained from the Royal College of Surgeons in Ireland Ethics Committee (REC1121) to collect blood from healthy consenting donors. Written informed consent was obtained from all donors before blood collection. Donors had refrained from taking acetylsalicylic acid, paracetamol or non-steroidal anti-inflammatory drugs in the previous 7 days. Venous blood was collected into S-Monovette 3.2% citrate tubes (Sarstedt) using a 20-gauge needle.

### Platelet preparation

#### Platelet-rich and platelet-poor plasma

Whole blood was centrifuged at 200 *g* for 20 min. The platelet rich plasma (PRP) supernatant was collected. The remaining red cells and buffy coat layer were further centrifuged at 1000 *g* for 10 min and the resulting platelet-poor plasma (PPP) supernatant was collected.

#### Washed platelets

As per^27^ whole blood was supplemented with 22% acid citrate dextrose (ACD, 120 mM sodium citrate, 110 mM glucose, 80 mM dextrose, pH 4.4) before centrifugation at 200 *g* for 20 min. The PRP supernatant was removed, and a further 10% ACD was added before centrifugation at 500 *g* for 15 minutes. After removal of the supernatant, the surface of the platelet pellet was washed gently three times with Krebs-Ringer Glucose buffer (120 mM NaCl, 4.9 mM KCl, 1.2 mM MgSO_4_, 1.7 mM KH_2_PO_4_, 8.3 mM Na_2_HPO_4_, 10 mM glucose, pH 7.4) supplemented with 0.01 U/ml grade 7 apyrase. The platelet pellet was then resuspended in this buffer to a concentration of 2 × 10^8^ platelets/ml and supplemented with 1 mM calcium chloride. Platelet counts were measured using a XQ-320 (Sysmex).

#### Aggregometry

Aggregation reactions were carried out at 37°C with 1200 rpm stirring on either a PAP-4 or PAP-8 aggregometer (Bio-data Corp). Traces were recorded for at least 10 minutes. A 1:9 bacteria:platelet volume ratio was used throughout. Platelets were incubated with the relevant inhibitor for at least 3 minutes prior to start of recording.

#### Dense granule secretion assay

90 μl of PRP was taken from the aggregation reaction at maximum aggregation or the corresponding time point and added to a white 96-well plate. 10 μl of Chrono-Lume^®^ was added to each sample and luminescence measured immediately on a Victor X plate reader.

#### Anti-*S. oralis* antibody ELISA

ATCC 10557 Sm^r^ and Fap1Janus were grown and washed as above before resuspension in PBS with 0.05% sodium azide. Protein concentrations were quantified using a Pierce^™^ BCA assay kit. 1 μg of the *S. oralis* preparations diluted in PBS was added per well to Nunc MaxiSorp 96 well plates and incubated at 4°C overnight. Plates were washed 3 times with PBS before blocking for 1 hr with blocking buffer (2% (w/v) BSA in PBS-0.1% Tween 20). Wash buffer (PBS-0.1% Tween 20) was used to wash plates 3 times before addition of serum diluted 1 in 40 in blocking buffer. Serum was serially diluted 1 in 4 to produce a 6-point dose response curve and incubated on plate for 1 hr. 100 μl total IgG mouse anti-human HRP-conjugated antibody (clone 2040–05, Southern Biotech) diluted 1:8000 in wash buffer was added to each well and incubated for 1 hr. 100 μl TMB-Core (Bio-Rad) was used to develop plate for up to 10 min, and the reaction stopped with 50 μl 0.2 M H_2_SO_4_. OD_450_ was read using a Victor X plate reader.

### Statistical analysis

All statistical analyses were conducted using GraphPad Prism 10.3.1. Shapiro-Wilk was carried out on data sets to establish normality. Where all data sets were parametric, for comparisons between two paired groups, or more than two paired groups, paired t-tests and repeated measures one-way ANOVA with Dunnett’s multiple comparisons were carried out respectively. Where one or more of the groups were non-parametric, Wilcoxon matched-pairs signed rank test and Friedman test with Dunn’s multiple comparisons test were carried out for two paired groups and more than two paired groups respectively. Data in bar graphs are presented as mean ± standard deviation or median ± interquartile range as stated in figure legends. *p* < .05 was considered significant.

## Results

### The infective endocarditis *S. oralis* isolate ATCC 10557 induces platelet aggregation

*S. oralis* strain ATCC 10557 was originally isolated from a patient with subacute bacterial endocarditis.^28^ To determine whether this strain could induce platelet aggregation, light transmission aggregometry was performed using platelet-rich plasma (PRP). Stimulation with ATCC 10557 Sm^r^ resulted in robust platelet aggregation, with a mean maximum light transmission of 77.1 ± 10.5%, comparable to that induced by the PAR-1 receptor agonist TRAP-6 (85.1% ± 8.0%) (Figure 1Ai).

Aggregation induced by ATCC 10557 Sm^r^ was preceded by a measurable lag phase, with a median lag time of 2.9 min (interquartile range [IQR] 2.0–7.0), significantly longer than that observed with TRAP-6 (median 0.05 min, IQR 0.03–0.07) (Figure 1Ai). Notably, donor responses were heterogeneous: 74% of donors exhibited rapid aggregation with lag times under 4 min, while the remaining 26% displayed delayed aggregation with lag times between 7 and 12 min. Given that lag time is often indicative of distinct aggregation mechanisms, subsequent mechanistic experiments focused on donors exhibiting rapid aggregation (<4 min) to ensure consistency.

To confirm platelet activation, dense granule secretion was assessed by measuring ATP release. Stimulation with ATCC 10557 Sm^r^ resulted in ATP release corresponding to 182.5% ± 17.3% of that induced by TRAP-6 (Figure 1Aii), demonstrating strong platelet activation.

### *S. oralis*-induced aggregation requires GPIIb/IIIa-dependent platelet signaling

To distinguish true platelet aggregation from agglutination, PRP was pre-incubated with the GPIIb/IIIa antagonist eptifibatide prior to bacterial stimulation. Eptifibatide completely abolished aggregation responses to ATCC 10557 Sm^r^ (Figure 1Bi), confirming that integrin αIIbβ3-dependent platelet signaling was required. Dense granule secretion was also fully inhibited under these conditions (Figure 1Bii). Similarly, treatment with the prostacyclin analogue PGE_1_ prevented aggregation (data not shown, *n* = 3), further confirming the requirement for platelet activation rather than passive crosslinking.

The contribution of secondary platelet activation pathways was next examined. Inhibition of cyclooxygenase (COX) using acetylsalicylic acid did not prevent aggregation but resulted in a mean increase in lag time of 2.5 min and an approximately 15% reduction in maximum aggregation (Figure 1Ci). Consistent with partial inhibition, ATP release was reduced to an average of 62% of control levels (Figure 1Cii). Inhibition of ADP signaling using the P2Y_12_ antagonist cangrelor had minimal effect on maximal aggregation in 4 of 5 donors, producing only a modest increase in lag time (<1 min) (Figure 1Di). However, ATP release was reduced by over 50% (Figure 1Dii), indicating a significant role for ADP in amplification of dense granule secretion rather than initiation of aggregation.

### *S. oralis* induces platelet aggregation via a Fap1-GP1bα interaction

Previous static binding assays have demonstrated that *S. oralis* ATCC 10557 binds to sialic acid on platelets via the SRRP Fap1.^25^ To determine whether Fap1 was required for platelet aggregation, PRP was stimulated with a Fap1-deficient mutant (ATCC 10557 Fap1 Janus). Loss of Fap1 abolished aggregation in 6 of 7 donors (Figure 2A). Aggregation traces were recorded for 10 min, corresponding to approximately four times the average lag time observed with the parental strain. In the single responding donor, aggregation occurred with a markedly prolonged lag time of 8 min, more than threefold longer than that induced by ATCC 10557 Sm^r^, suggesting engagement of an alternative, less efficient mechanism.

A conserved threonine–arginine motif within SRRPs has been shown to be critical for efficient platelet binding.^25,29^ Mutation of the corresponding arginine residue (R489E) in Fap1 completely abolished platelet aggregation in all donors tested (Figure 2B), confirming the importance of this motif for functional platelet engagement.

Given that GP1bα contains approximately 80% of the total platelet sialic acid content,^30,31^ we next assessed its role directly. Preincubation of PRP with the anti-GP1bα binding antibody AK2 abolished aggregation in 6 of 7 donors (Figure 2Ci). Dense granule secretion in non-aggregating donors was reduced to an average of 12% of control levels (Figure 2Cii). In the remaining donor, AK2 did not fully prevent aggregation but increased the lag-time, from 1.3 min to 5 mins (data not shown), indicating partial dependence on GPIbα engagement.

### A plasma component is required to cause *S. oralis*-induced aggregation in most donors

To determine whether the Fap1-GPIbα interaction was sufficient to induce aggregation, experiments were repeated using washed platelets to eliminate plasma components. In contrast to responses observed in PRP, ATCC 10557 Sm^r^ failed to induce aggregation in washed platelets from 7 of 9 donors (Figure 3), indicating a requirement for an additional plasma-derived factor in the majority of individuals. In the two donors whose washed platelets did aggregate, lag times were prolonged (4.0 and 5.1 min) relative to responses in PRP (Figure 3), suggesting reduced efficiency of activation in the absence of plasma.

### Addition of antibody reinstates *S. oralis*-induced aggregation in washed platelets

Given previous evidence implicating IgG in *S. oralis*-induced platelet aggregation,^21^ pooled human IgG (hIgG) was added to washed platelets prior to stimulation. Supplementation with hIgG fully restored aggregation responses to levels comparable to those observed in PRP (Figure 4A). Notably, lag times in washed platelets supplemented with hIgG were shorter than those observed in WP in the two donors who aggregated in washed platelets without IgG.

Aggregation remained dependent on GPIIb/IIIa signaling, as eptifibatide completely inhibited responses in hIgG-supplemented washed platelets (Figure 4B). To confirm the involvement of FcγRIIA, platelets were pre-incubated with the FcγRIIA-blocking antibody IV.3. Blockade of FcγRIIA abolished aggregation in both PRP (Figure 4Ci) and washed platelets supplemented with hIgG (Figure 4Ciii). Dense granule secretion in PRP was also completely inhibited by IV.3 (Figure 4Cii).

A dose–response analysis revealed that IgG concentration strongly influenced aggregation kinetics within donors. Decreasing hIgG levels resulted in reduced maximal aggregation and progressively longer lag times (Figure 4D). Median lag time increased nearly fivefold, from 1.5 min at 100 μg/ml hIgG to 7.4 min at 6.25 μg/ml. At lower IgG concentrations, marked inter-donor variability was observed, with some donors retaining near-maximal aggregation while others failed to aggregate entirely.

### Addition of IgG does not bypass the requirement for Fap1-GP1bα interaction

To determine whether IgG alone could compensate for loss of Fap1-mediated adhesion, washed platelets supplemented with hIgG were stimulated with Fap1-deficient strains. Addition of hIgG failed to restore aggregation in response to either ATCC 10557 Fap1 Janus or Fap1R489E (Figure 5A), even when assays were extended to 20–30 min.

To confirm strain-specific IgG binding was not responsible for the difference in aggregation between ATCC 10557 Smr and Fap1 Janus, total IgG titers against the two strains were measured by ELISA and found to be comparable (Supplementary Figure S1). Finally, washed platelets from donors whose platelets aggregated to the parental strain in the absence of IgG also failed to respond to Fap1-deficient mutants (Figure 5B), confirming that Fap1–GPIbα engagement is a prerequisite for platelet aggregation, irrespective of IgG availability.

## Discussion

*Streptococcus oralis* is increasingly recognized as a major causative agent of infective endocarditis, yet the platelet activation mechanisms underpinning its pathogenicity have remained incompletely defined. In this study, we demonstrate that an IE-derived *S. oralis* isolate induces potent platelet aggregation through a dual receptor mechanism requiring both Fap1-mediated engagement of GPIbα and antibody-dependent signaling via FcγRIIA (Figure 6). Our data support a model in which direct bacterial adhesion to platelets provides a necessary anchoring step, enabling IgG-mediated crosslinking of FcγRIIA and downstream integrin αIIbβ3 activation, culminating in robust aggregation and dense granule secretion.

The involvement of GPIbα in bacterial-induced platelet activation is well established for several pathogens, including *S. sanguinis*^9,19^ and *S. gordonii*.^10^ Likewise, multiple different bacterial strains utilize FcγRIIA to induce platelet aggregation.^27,32–36^ Previous research from our group showed GP1bα interaction is necessary for platelets to adhere to *S. oralis* under shear, with involvement from FcγRIIA required during light transmission aggregometry experiments.^22^ The only two other strains shown to utilize both GPIbα and IgG/FcγRIIA interactions are *H. pylori* and *P. gingivalis*.^37,38^ Here we provide the first evidence that *S. oralis* requires coordinated signaling through both receptors to induce platelet aggregation in the majority of donors. Importantly, our findings establish Fap1 as the critical bacterial adhesin responsible for initiating this process. Disruption of Fap1 function, either through gene deletion or mutation of a conserved sialic acid-binding motif, completely abrogated platelet aggregation, even in the presence of exogenous IgG. This indicates that antibody-dependent FcγRIIA signaling alone is insufficient to drive platelet activation in the absence of initial Fap1–GPIbα engagement.

Bacterial-induced platelet aggregation is characteristically associated with a lag phase, reflecting the time required for receptor engagement, signaling amplification, and integrin activation.^17^ While lag time has traditionally been viewed as a strain-specific property, our data reveal substantial inter-individual variability in platelet responses to a single *S. oralis* strain. Although most donors exhibited rapid aggregation, two differing subgroups were identified in this study: one subgroup who did not require plasma-derived factors for aggregation to *S. oralis* to occur, and another subgroup who displayed delayed responses in PRP. These observations suggest that host-specific factors strongly influence the efficiency of platelet activation and may contribute to differential susceptibility to vegetation formation during transient bacteremia.

Our washed platelet experiments demonstrate that IgG is a critical determinant of aggregation efficiency, with both the magnitude and kinetics of platelet activation strongly influenced by antibody concentration. This finding aligns with previous reports implicating IgG in platelet responses to oral streptococci^22^ and extends them by demonstrating a clear dose-dependent effect. IgG supplementation led to faster aggregation in the two donors whose platelets aggregated washed platelets alone, suggesting that antibody-mediated FcγRIIA engagement can accelerate platelet activation once bacterial adhesion has occurred.

The requirement for IgG raises intriguing questions regarding the impact of host immune history on IE risk. Although not directly examined here, IgG titers following streptococcus exposure are known to remain elevated for prolonged periods.^39^ Levels of anti-*S. oralis* antibody may be further boosted by repeated systemic exposure through poor oral hygiene or chronic gingival inflammation, consequently lowering the threshold for platelet activation upon bacteremia. In individuals with preexisting cardiac lesions or prosthetic valves, such enhanced platelet responsiveness could favor vegetation formation and bacterial persistence. Conversely, individuals with lower antibody titers or reduced FcγRIIA signaling capacity may be relatively protected, despite similar levels of bacterial exposure. This raises the possibility that thrombotic risk in IE is not static, but fluctuates with host immune status, and suggests that antibody titer could represent a previously underappreciated determinant of susceptibility. Prospective clinical studies examining platelet reactivity and antibody kinetics in patients with confirmed streptococcal infection will be required to address this directly.

Genetic variability in platelet receptors may further modulate these responses. Copy number variation^40^ and polymorphisms within FcγRIIa are known to influence IgG binding affinity,^41,42^ while polymorphisms in GPIbα affect both receptor abundance^43^ and glycosylation patterns.^44–47^ These host factors could alter the balance between direct bacterial adhesion and antibody-dependent signaling, contributing to the heterogeneous platelet phenotypes observed in this study. Although genetic analyses were beyond the scope of the present work, our findings provide a strong rationale for future investigations linking platelet genotype to IE susceptibility.

The broader relevance of this mechanism is underscored by the presence of homologous serine-rich repeat proteins in other oral streptococci implicated in IE. Adhesins such as GspB, Hsa, and SrpA similarly target sialylated platelet glycoproteins, suggesting that coordinated engagement of GPIbα and FcγRIIA may represent a conserved strategy by which oral streptococci exploit platelet biology to promote intravascular colonization. Targeting these early platelet–bacterium interactions may therefore represent an attractive therapeutic or prophylactic strategy. It is important to consider that while oral streptococci are strongly associated with sub-acute IE, increasingly epidemiological studies are identifying *S. aureus* as the most common single IE pathogen.^48,49^ We therefore view *S. oralis* not as the sole major culprit in IE, but as a relevant and mechanistically informative oral streptococcal pathogen whose platelet-interaction strategy may illuminate broader principles of vegetation formation. To this end, Figure 7 summarizes the major relevant IE causing pathogens, and their shared platelet activation pathways.

An important limitation of the present study is that platelet responses were examined in PRP and washed platelets rather than whole blood. Whilst this allowed detailed mechanistic platelet-bacterial interactions to be uncovered, it does not take into account additional interactions from surrounding blood components and endothelial cells. Neutrophil extracellular traps have been implicated in vegetation formation in experimental IE, where they enhance and stabilize bacterial-platelet aggregates,^52^ potentially amplifying the platelet aggregation pathway described, making them another attractive target to study. We propose our described Fap1-GP1bα/FcγRIIA pathway as one component of a broader thromboinflammatory process, positioned at a key early step in vegetation initiation and propagation.

Furthermore, our use of healthy donors, while allowing us to define a core mechanism of *S. oralis*-induced platelet activation in the absence of major clinical confounders, may not fully reflect platelet behavior in hospitalized or comorbid patients. Future work comparing platelet responses across healthy and high-risk patient populations will be required to determine whether comorbidity-associated platelet priming amplifies the Fap1-GP1bα/FcγRIIA pathway described in this study.

Our findings could however have implications for ongoing debates surrounding antibiotic prophylaxis for dental procedures. Current guidelines vary internationally, with some recommending targeted prophylaxis for high-risk individuals and others advising against routine use.^53^ The marked inter-individual variability observed here suggests that host-specific factors, such as platelet receptor polymorphisms or anti-streptococcal antibody levels, could help identify individuals at heightened risk of platelet-driven vegetation formation. Incorporating such biomarkers into clinical risk stratification frameworks could enable more precise, evidence-based prophylactic strategies while minimizing unnecessary antibiotic exposure.

## Conclusion

In conclusion, we demonstrate that an infective endocarditis isolate of *Streptococcus oralis* activates platelets through a dual, non-redundant mechanism requiring Fap1-mediated engagement of glycoprotein Ibα and antibody-dependent signaling via FcγRIIA. Direct bacterial adhesion is an essential initiating event that enables IgG-driven platelet activation, integrin αIIbβ3 signaling, and robust aggregation. This pathway exhibits substantial inter-individual variability, reflecting differences in host immune and platelet receptor biology, and may help explain why only a subset of individuals exposed to oral streptococci develop infective endocarditis.

By defining how *S. oralis* exploits platelet receptors to promote thrombus formation, this work provides new insight into the earliest events of endocardial colonization and identifies platelet–bacterium interactions as potential targets for risk stratification and therapeutic intervention. As antibiotic resistance continues to challenge existing preventative strategies, understanding host-specific determinants of platelet activation may enable more precise approaches to infective endocarditis prevention in high-risk populations.

## Supplementary Material

Supp 1

Supplemental data for this article can be accessed online at https://doi.org/10.1080/09537104.2026.2688088.

## Figures and Tables

**Figure 1.**
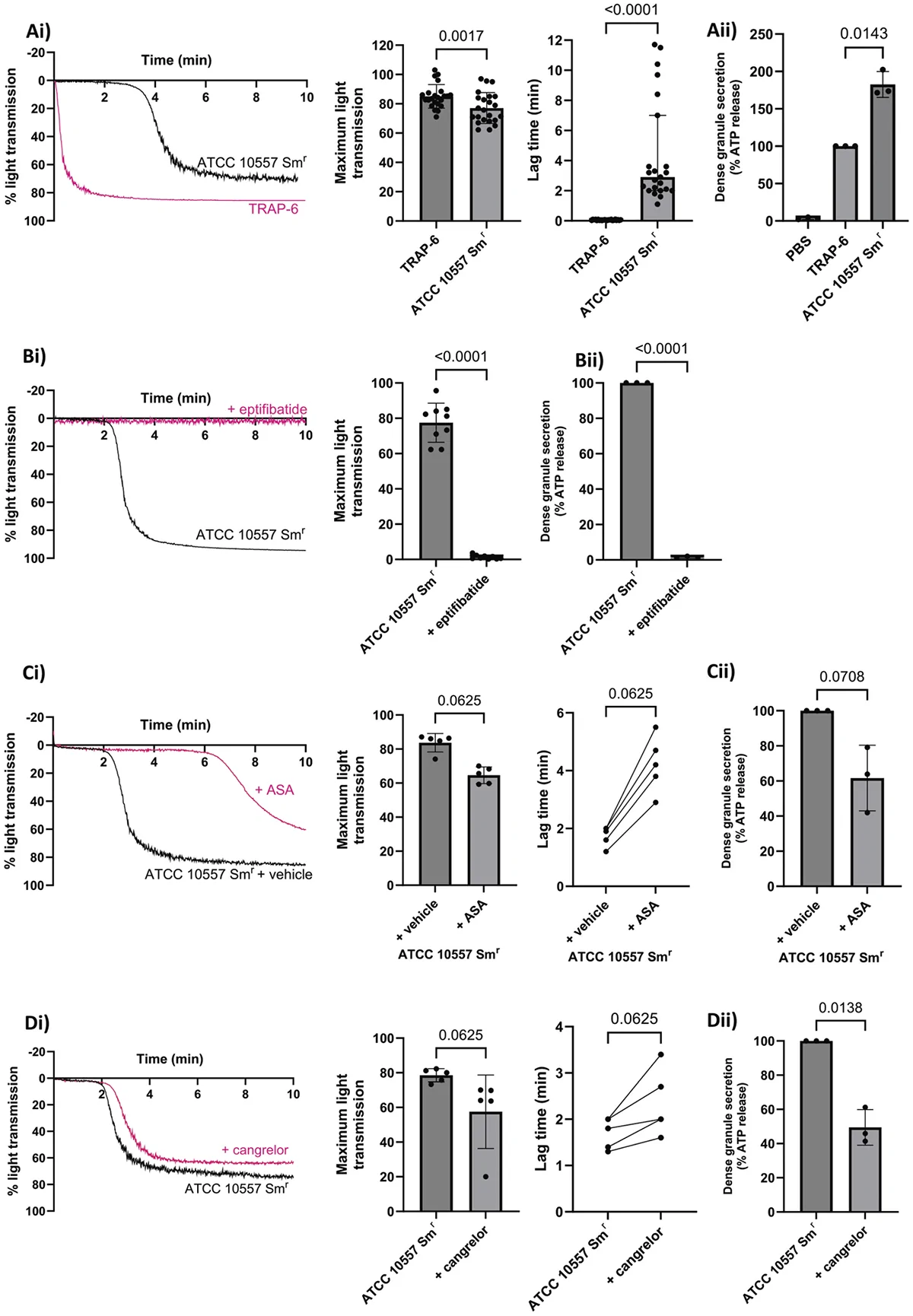
An infective endocarditis isolate of *Streptococcus oralis* induces platelet aggregation and dense granule secretion. PRP was stimulated with *S. oralis* ATCC 10557 Sm^r^ (OD_600_ = 1.0; 9:1 platelet:bacteria volume ratio) or with 20 μM TRAP-6 as a positive control. (Ai) Representative aggregation traces (left), maximum light transmission (middle; mean ± SD), and lag times (right; median ± interquartile range) are shown (*n* = 23). Lag time is defined as the interval from agonist addition to the inflection point marking the onset of aggregation. (Aii) Dense granule secretion was assessed by measuring ATP release using a luciferin–luciferase assay. Data are normalized to ATP release induced by TRAP-6 (mean ± SD, *n* = 3). (Bi) PRP was pre-incubated with the GPIIb/IIIa antagonist eptifibatide (9 μM) prior to stimulation with *S. oralis*. Representative trace (left) and quantification of maximum aggregation (right) are shown (*n* = 9). (Bii) ATP release corresponding to (Bi), normalized to vehicle-treated *S. oralis* stimulation (mean ± SD, *n* = 3). (Ci) PRP was pre-treated with acetylsalicylic acid (ASA,100 μM) or vehicle control before bacterial stimulation. Representative trace (left), maximum aggregation (middle), and lag time (right) are shown (*n* = 5). (Cii) ATP release from samples in (Ci), normalized to *S. oralis*-stimulated controls (mean ± SD, *n* = 3). (Di) PRP was pre-treated with the P2Y_12_ antagonist cangrelor (1 μM). Representative trace (left), maximum aggregation (middle), and lag time (right) are shown (*n* = 5). (Dii) ATP release corresponding to (Di), normalized to *S. oralis*-stimulated controls (mean ± SD, *n* = 3).

**Figure 2.**
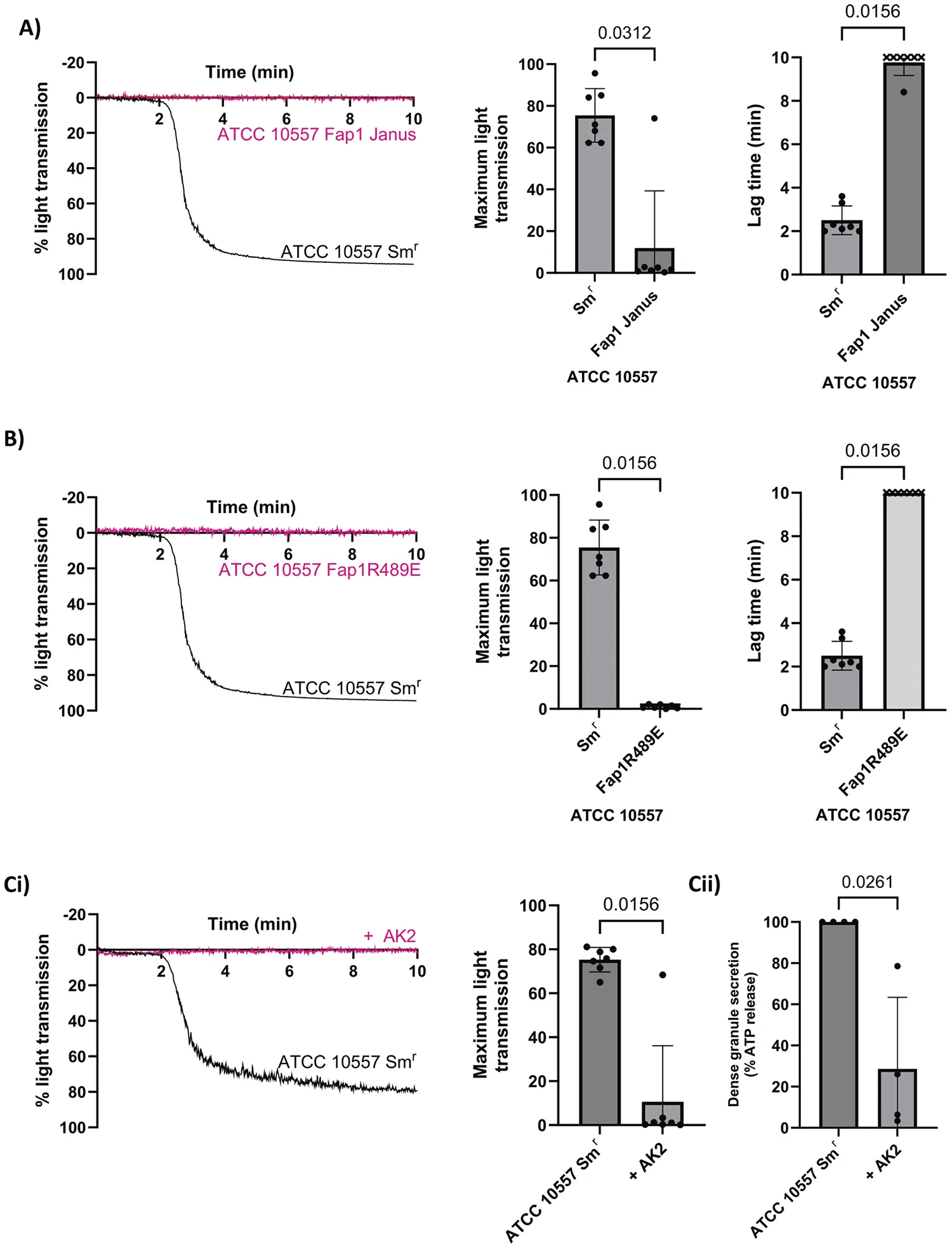
*S. oralis*-induced platelet aggregation requires Fap1-mediated engagement of platelet GPIbα. PRP was stimulated with *S. oralis* strains (OD_600_ = 1.0; 9:1 platelet:bacteria volume ratio). (A) Aggregation induced by the parental strain ATCC 10 557 Sm^r^or the Fap1-deficient mutant (Fap1 Janus). Representative trace (left), maximum aggregation (middle), and lag times (right) are shown (*n* = 7). (B) Aggregation induced by ATCC 10557 Sm^r^ or the Fap1 R489E point mutant. Representative trace (left), maximum aggregation (middle), and lag times (right) are shown (*n* = 7). (Ci) PRP was pre-incubated with an anti-GPIbα monoclonal antibody (AK2, 10 μg/ml) or vehicle control prior to stimulation with ATCC 10557 Sm^r^. Representative trace (left) and maximum aggregation (right) are shown (*n* = 7). (Cii) ATP release corresponding to (Ci), normalized to vehicle-treated *S. oralis* stimulation (mean ± SD, *n* = 4). Where no aggregation occurred, an “×” is plotted at 10 min on lag-time graphs.

**Figure 3.**
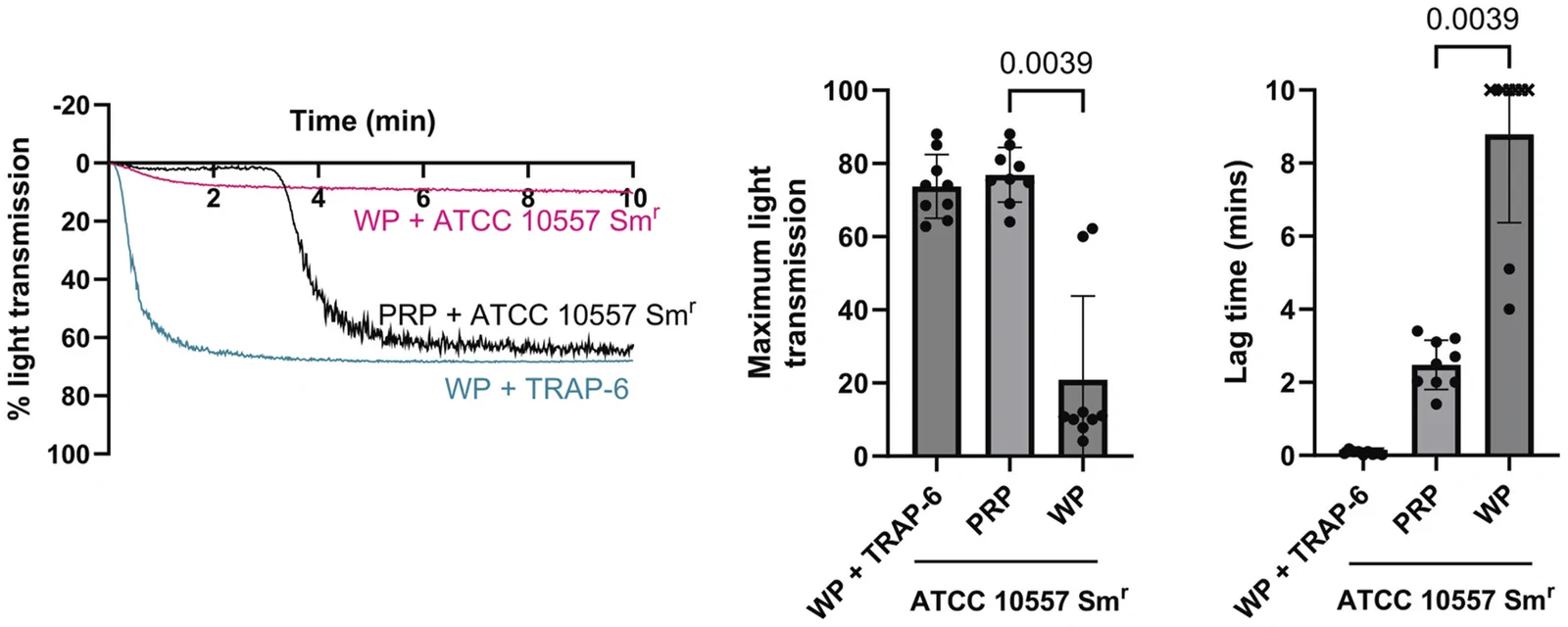
A plasma component is required for *S. oralis*-induced platelet aggregation in most donors. Aggregation responses of PRP and washed platelets (WP) to *S. oralis* ATCC 10557 Sm^r^ (OD_600_ = 1.0; 9:1 ratio) were compared. TRAP-6 (20 μM) was used as a positive control for WP. Representative traces (left), maximum aggregation (middle), and lag times (right) are shown (*n* = 9). Where no aggregation occurred, an “×” is plotted at 10 min.

**Figure 4.**
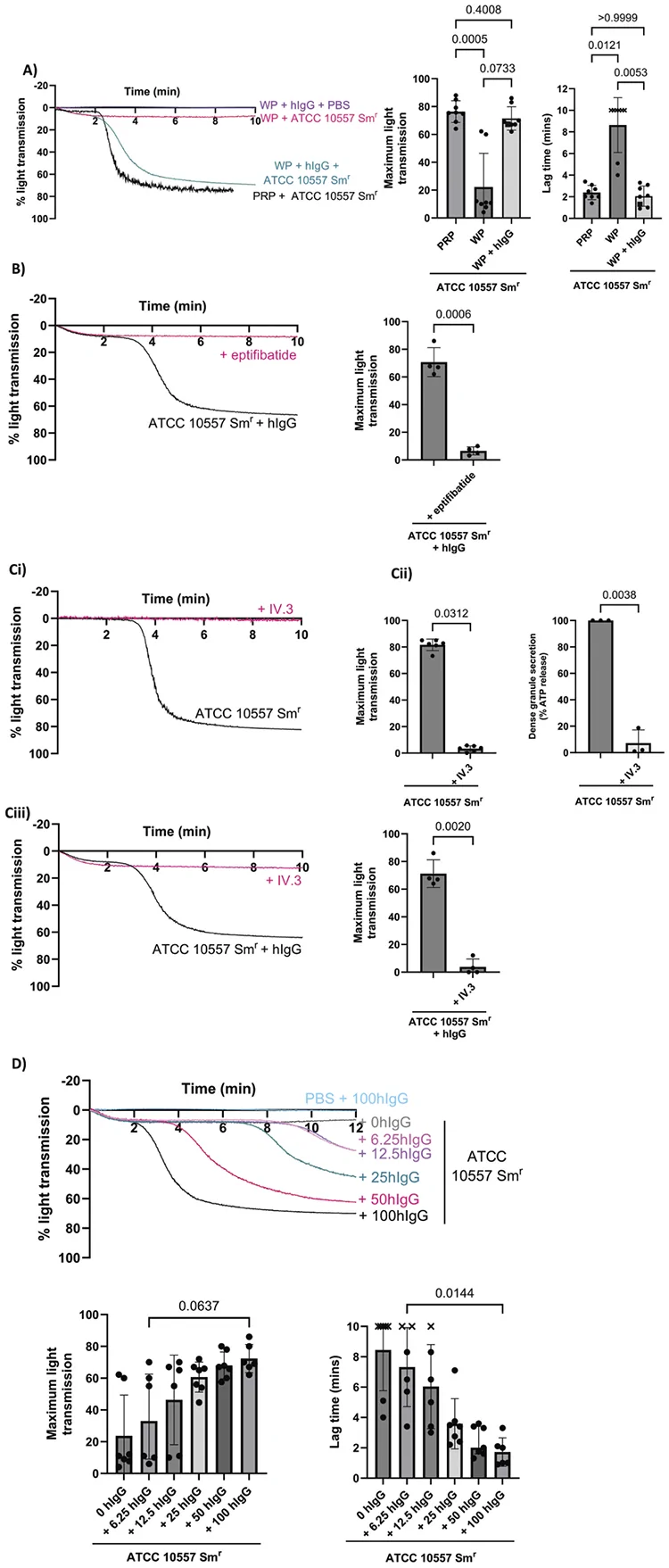
IgG restores *S. oralis*-induced platelet aggregation in washed platelets via FcγRIIA. PRP, washed platelets (WP), or WP supplemented with pooled human IgG (hIgG; 50–100 μg/ml) were stimulated with *S. oralis* ATCC 10557 Sm^r^ (OD_600_ = 1.0; 9:1 ratio). (A) Representative traces (left), maximum aggregation (middle), and lag times (right) comparing PRP, WP, and WP + hIgG (*n* = 8). (B) WP supplemented with hIgG were pre-treated with eptifibatide (9 μM) prior to bacterial stimulation. Representative trace (left) and maximum aggregation (right) are shown (*n* = 4). (Ci) PRP was pre-incubated with an FcγRIIA-blocking antibody (IV.3, 10 μg/ml). Representative trace (left) and maximum aggregation (right) are shown (*n* = 6). (Cii) ATP release corresponding to (Ci), normalized to vehicle-treated *S. oralis* stimulation (mean ± SD, *n* = 3). (Ciii) WP supplemented with hIgG were pre-treated with IV.3 prior to stimulation. Representative trace (left) and maximum aggregation (right) are shown (*n* = 4). (D) Dose–response of hIgG (6.25–100 μg/ml) in WP stimulated with *S. oralis*. Representative traces (top), maximum aggregation (bottom left), and lag times (bottom right) are shown (*n* = 6). Where no aggregation occurred, an “×” is plotted at 10 min.

**Figure 5.**
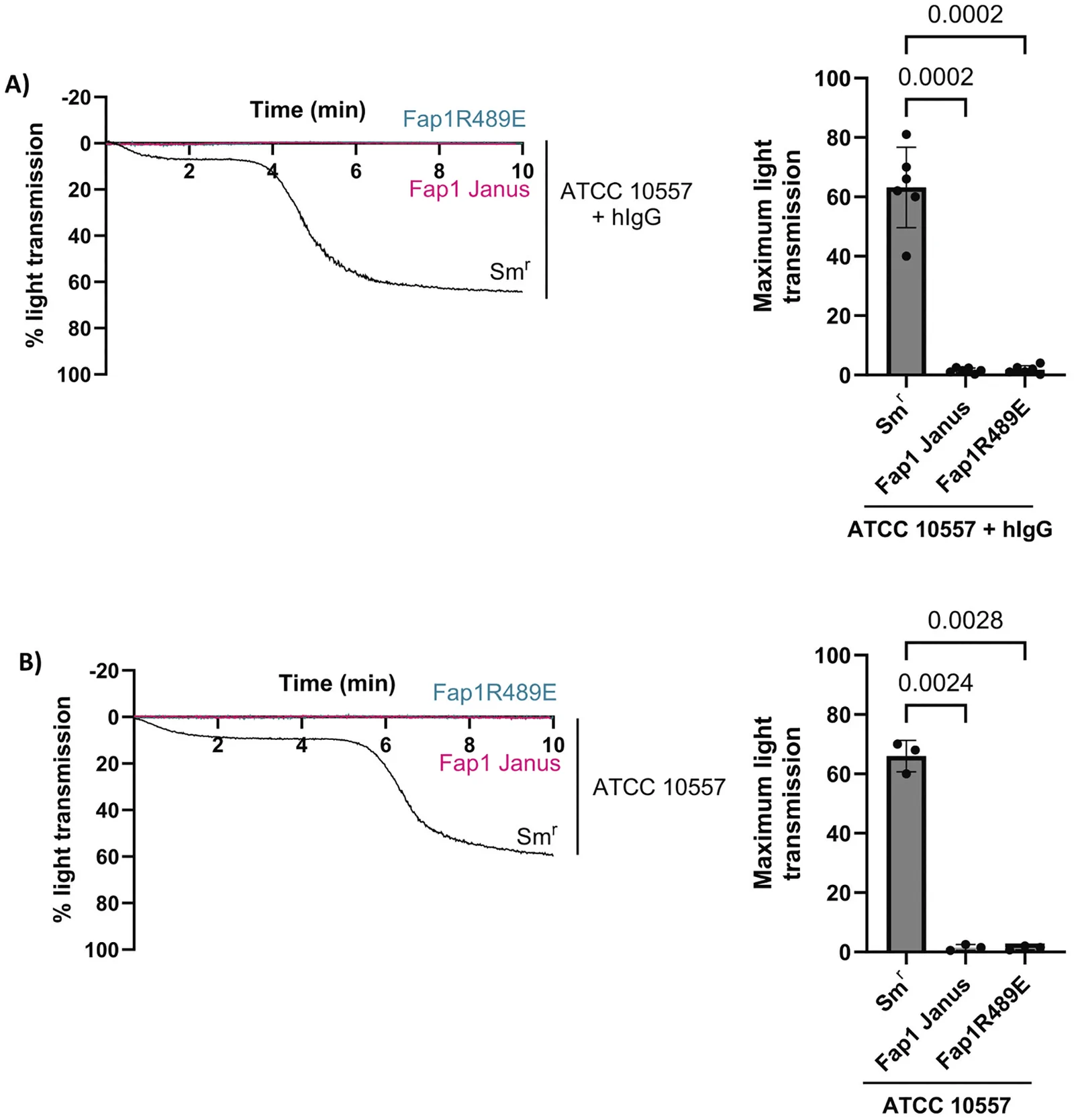
IgG does not bypass the requirement for Fap1–GPIbα interaction. Washed platelets (WP) supplemented with hIgG (50–100 μg/ml; all donor platelets were supplemented with 100 μg/ml except one donor whose platelets aggregated with the addition of 100 μg/ml, so 50 μg/ml was used to avoid spontaneous aggregation) were stimulated with ATCC 10557 Sm^r^, Fap1 Janus, or Fap1R489E strains. (A) Representative traces (left) and maximum aggregation (right) are shown (*n* = 6). (C) WP (without added IgG) from responsive donors were stimulated with parental or Fap1-deficient strains. Representative traces (left) and maximum aggregation (right) are shown (*n* = 3).

**Figure 6.**
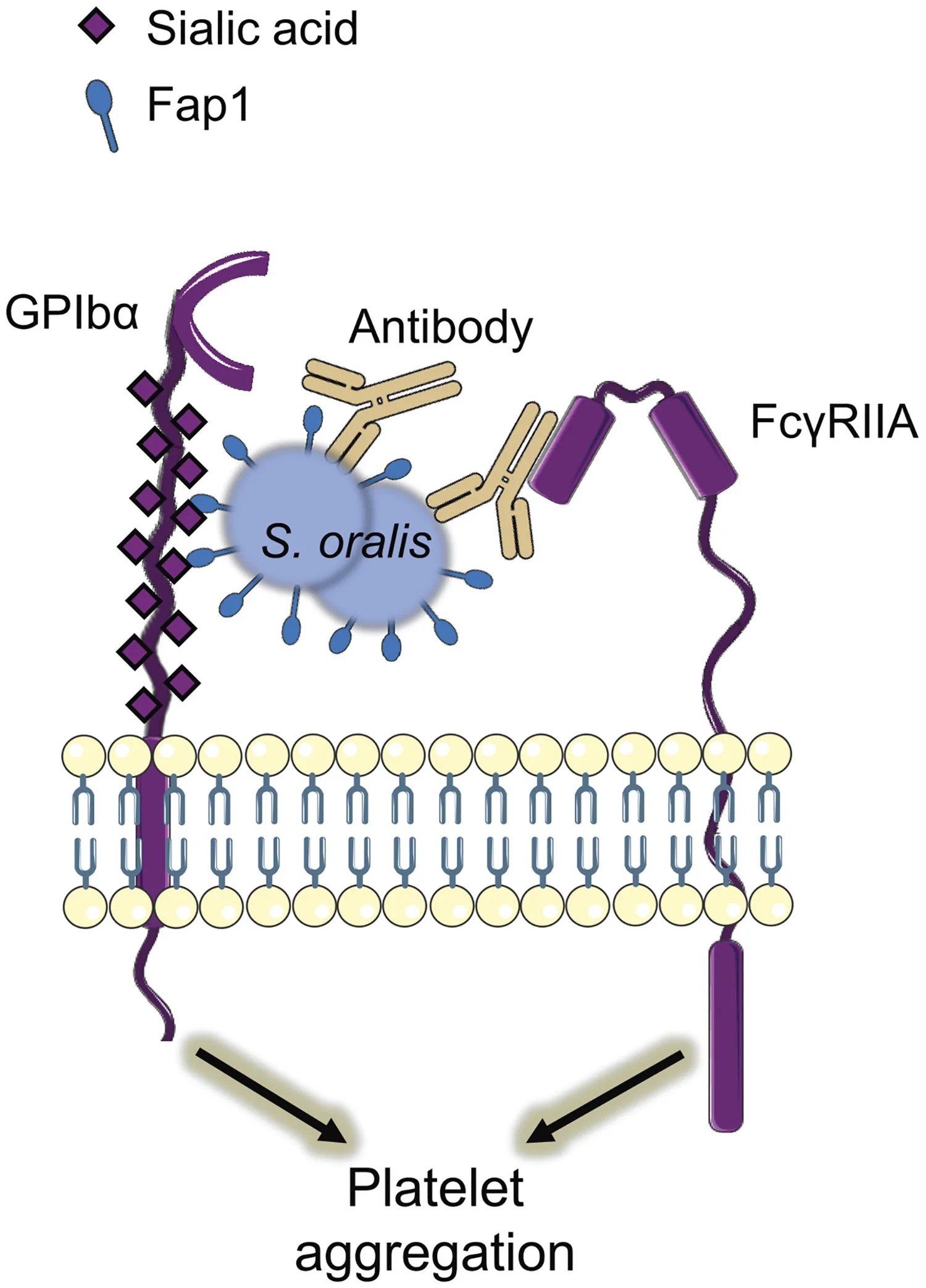
Proposed mechanism of *S. oralis*-induced platelet aggregation. Schematic representation of the proposed dual mechanism by which *S. oralis* induces platelet aggregation. Fap1 mediates bacterial adhesion to sialylated glycoprotein Ibα on the platelet surface, providing an essential anchoring step that enables IgG binding to FcγRIIA and FcγRIIA crosslinking. Coordinated signaling through GPIbα and FcγRIIA leads to integrin GPIIb/IIIa activation, dense granule secretion, and robust platelet aggregation.

**Figure 7.**
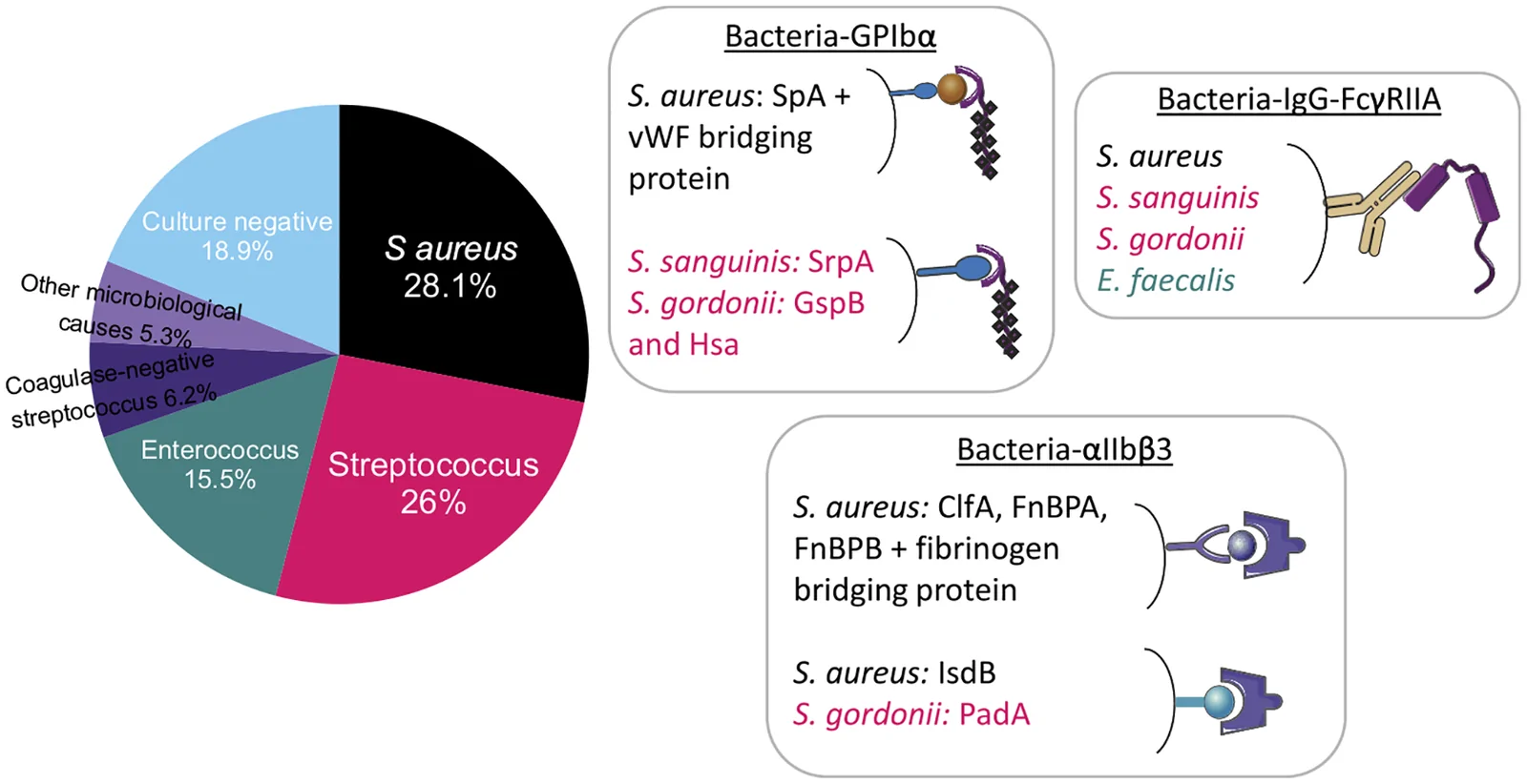
Shared bacteria-platelet interaction pathways in IE pathogens. Proportions of IE causative pathogens vary slightly by world region. For the purposes of this figure, percentages from^50^ were used. GPIbα engagement, IgG-FcγRIIA interactions, and αIIbβ3 activation are key pathways in multiple IE causing bacteria.^51^ a short summary of these occurring in known IE causing bacteria is provided – bacterial strains are color coded to match the appropriate portion of the pie chart. Specific interacting bacterial proteins are listed where known.
